# Functional Characteristics Derived from the Structural Design of Bispecific Antibodies

**DOI:** 10.3390/ph19081245

**Published:** 2026-08-07

**Authors:** Jaehee Han, Su Yeon Lim, Yeongbeom Kim, Deokhwa Jeong, Hyun-Ouk Kim, Suk-Jin Ha, Jeong-Ann Park, Young-Wook Won, Kwang Suk Lim

**Affiliations:** 1 Department of Smart Health Science and Technology, Kangwon National University, Chuncheon 24341, Republic of Korea; gkswogml03@kangwon.ac.kr (J.H.); suyeonlim9846@kangwon.ac.kr (S.Y.L.); rladudqja215@gmail.com (Y.K.); 93deokgoo@kangwon.ac.kr (D.J.); kimhoman@kangwon.ac.kr (H.-O.K.); sjha@kangwon.ac.kr (S.-J.H.); 2 Department of Biomedical Engineering, College of Engineering, University of North Texas, Denton, TX 76203-5017, USA; youngwook.won@unt.edu; 3 Department of Biotechnology and Bioengineering, College of ACE, Kangwon National University, Chuncheon 24341, Republic of Korea; 4 Institute of Fermentation and Brewing, Kangwon National University, Chuncheon 24341, Republic of Korea; 5 Department of Environmental Engineering, College of ACE, Kangwon National University, Chuncheon 24341, Republic of Korea; pjaan@kangwon.ac.kr

**Keywords:** bispecific antibody, structural architecture, valency, epitope geometry, affinity asymmetry, immune cell redirection

## Abstract

Bispecific antibodies (bsAbs) are engineered to recognize either two distinct antigens or two different epitopes on the same antigen within a single molecule. This design varies according to the intended indication and mechanism of action; the factors considered during design are critical determinants of antigen binding, pharmacological activity, productivity, and safety. In this review, bsAbs are classified into fragment-based formats and Fc-containing IgG-like formats, with the latter further divided into symmetric and asymmetric architectures. Based on this structural framework, we discuss how key design parameters—including valency, epitope geometry, affinity and binding kinetics, and linker architecture—influence avidity, immune synapse formation, receptor clustering, signaling modulation, and toxicity profiles. We further compare preclinical and clinical examples across representative target combinations, including CD19 × CD3, CD20 × CD3, BCMA × CD3, HER2 × HER2, and EGFR × MET, to illustrate how different molecular formats yield distinct therapeutic outcomes even when the target combinations are similar. By linking structural classification with mechanism-based interpretation and within-target comparisons, this framework relates individual design variables directly to their preclinical and clinical consequences. Finally, we describe how the energy-based molecular modeling platform Rosetta and the deep-learning-based structure-prediction system AlphaFold are applied to support interface optimization, chain-pairing control, epitope geometry prediction, and structure-guided candidate prioritization. Overall, this review can provide a structure–function framework for bsAb design by integrating key structural determinants, their functional consequences, and emerging AI-based predictive strategies to facilitate the selection of optimal molecular architectures for specific therapeutic applications.

## 1. Introduction

Antibody therapies have addressed the limitations of nonspecific systemic toxicity associated with conventional chemotherapy in various diseases, including cancer, infectious diseases, and autoimmune diseases. This effect is based on their specificity for target antigens [1]. Early antibody therapies were developed as monoclonal antibodies (mAbs), which bind specifically to a single antigen. However, several limitations were reported during clinical application. These included reduced therapeutic responses due to tumor antigen heterogeneity and treatment resistance caused by activation of compensatory signaling pathways [1,2,3]. mAbs also have limitations in directly bridging or activating adaptive immune cells, such as T cells. Accordingly, bispecific antibodies (bsAbs), which recognize two antigens simultaneously, have been developed. bsAbs have attracted attention as a strategy to improve tumor targeting and enhance immune cell-mediated antitumor responses [1,2,3,4].

bsAbs are designed to simultaneously recognize two different target antigens or two different epitopes on the same antigen [4,5]. Unlike conventional mAbs, bsAbs have been utilized for immune cell redirection and dual receptor blockade within a single molecule [1,2,3,4,5]. Factors determining the three-dimensional molecular binding structure should be considered, in addition to simple target combinations, for bispecific antibodies to function in accordance with the intended mechanism [4,6,7]. Valency, epitope geometry, affinity and binding kinetics, linker length and flexibility, and Fc-region arrangement have been especially recognized as key factors that directly influence immunological synapse stability, receptor clustering, tissue selectivity, and *in vivo* toxicity [4,6,7,8,9].

Thus, in bsAb development, a precise understanding of the relationship between molecular structure and function is required [4,6,7]. Over the past decade, more than 100 bsAb platforms have been proposed, ranging from fragment-based formats to symmetric and asymmetric IgG-like structures [4,5]. In clinical studies, many bsAbs have also shown meaningful outcomes in hematological malignancies, solid tumors, ophthalmic diseases, and autoimmune diseases [1,2,3]. In addition, recent advances in artificial intelligence (AI) have accelerated the utilization of computational design and antigen–antibody binding prediction technologies. Following these trends, recent attempts to transition from research based on iterative design and experimental validation to design based on structure prediction have been reported. Rosetta is an energy-based molecular modeling platform used to evaluate and redesign protein interfaces, whereas AlphaFold is a deep-learning-based system used to predict the three-dimensional structures of proteins and protein complexes. In bsAb development, these tools can support chain-pairing optimization, interface evaluation, epitope-geometry analysis, and structure-guided candidate prioritization [10,11,12,13].

In this review, we systematically classify bsAbs developed to date according to their molecular structural architectures. We also organize the structure–function relationships with a focus on valency, epitope geometry, binding affinity and kinetics, and linker design. Based on these principles, molecules sharing the same target combination are compared to examine how differences in molecular architecture are associated with distinct functional, pharmacokinetic, and therapeutic outcomes. This organization connects format-level classification with mechanism-based interpretation and the subsequent evaluation of preclinical and clinical examples. Furthermore, we examine how recent Rosetta and AlphaFold-based in silico design strategies are expanding the understanding and application of these structure–function correlations and explore the future directions of bsAb development.

## 2. Structural Classification of Bispecific Antibodies

bsAbs are broadly classified into fragment-based structures and IgG-like structures [4,5]. These structural formats are known to affect pharmacological performance, expression stability, and pharmacokinetic properties [4] (Table 1).

### 2.1. Fragment-Based Bispecific Antibody Structures

Fragment-based bsAbs are designed by combining only the variable domains responsible for antigen recognition, thereby omitting the Fc region. Therefore, the key design factor is how antigen-binding modules for each antigen are assembled.

These structures have functional advantages and limitations arising from the absence of the Fc region. Fragment-based bsAbs offer structural simplicity and low molecular weight. Low molecular weight provides potential advantages for tissue penetration and access to the tumor microenvironment [14]. In contrast, half-life extension through FcRn-mediated recycling is difficult, and Fc-dependent effector functions, such as ADCC and CDC, are limited [4,5]. Therefore, complementary strategies, such as continuous intravenous infusion or fusion with carrier proteins, were used in clinical applications [27,28,29].

**Figure 1:**
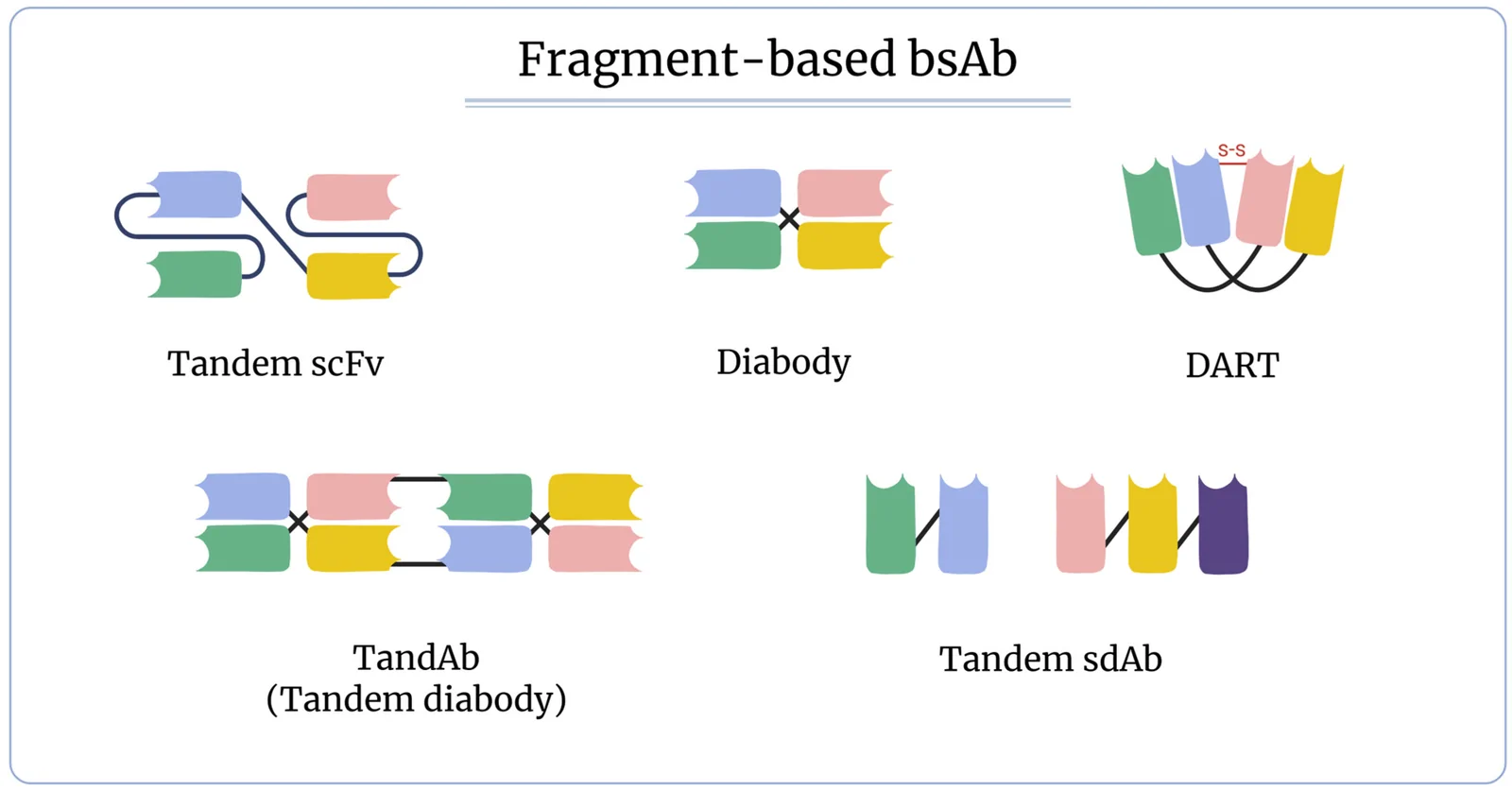
Structural architectures of fragment-based bispecific antibody platforms. Representative Fc-free bsAb formats, including tandem scFv, diabody, DART, TandAb, and sdAb-based structures, are shown. These architectures differ in chain organization, valency, linker dependence, and molecular size. bsAb (Bispecific Antibody); scFv (Single-Chain Variable Fragment); DART (Dual-Affinity Re-Targeting); sdAb (Single-Domain Antibodies).

Representative examples of this structural design include single-chain variable fragments (scFvs), diabodies, tandem diabodies, and single-domain antibodies (sdAbs) [14,30,31,32,33] (Figure 1).

#### 2.1.1. Tandem scFv

A tandem scFv is a single polypeptide structure in which two scFvs are linked in series by a flexible linker [14]. This single chain avoids heavy/light chain mispairing, which could occur in IgG-like formats, offering a production advantage [4]. In this structure, the spatial arrangement of the two antigen-binding modules was reported to be influenced by the order of the scFvs and by the length and flexibility of the linker [34,35].

#### 2.1.2. Diabody Formats

A diabody is a dimeric structure formed by restricting intrachain VH–VL pairing with a short linker and inducing interchain VH–VL pairing between two different chains. Its design is based on VH1–VL2 and VH2–VL1 combinations. This interchain pairing was demonstrated by Holliger et al. to fix the relative arrangement between domains in a more constrained manner than tandem scFv [31]. Therefore, the antigen-binding efficiency of this structure could depend not only on the inter-antigen distance but also on the orientation in which each antigen presents its epitope on the cell surface [7,8].

DART (Dual-Affinity Re-Targeting) is an improved diabody-derived format in which a disulfide bond is introduced between the two chains. This structure aims at improving structural stability and molecular homogeneity. It has been viewed as an approach to improve assembly stability and production reproducibility while maintaining the functional properties of a diabody [36].

A tandem diabody (TandAb) is a bispecific format containing tetravalent variable domains generated by linking two diabodies in series. This structural feature provides multivalent binding properties similar to those of a typical 2 + 2 format. Increased antigen-binding strength was confirmed by comparison with early diabody formats [30]. However, due to effects on pharmacokinetic properties and tissue penetration, optimization of the molecular weight range and structural complexity has been required depending on the application context [5,30].

#### 2.1.3. Single-Domain Antibody (sdAb)-Based Structures

Single-domain antibody (VHH or VH)-based bsAbs are concatenated with two or more single-variable domains. Heavy/light chain-pairing problems during Fab assembly are avoided by the absence of the VL domain [32,33]. Small size and high modularity facilitate multidomain combinatorial design. These properties could enhance applicability to targets with limited epitope accessibility. The variation in binding efficiency in this structure depends on the location and structural constraints of the target epitope. Moreover, its thermal stability and tendency to aggregate also depend on the properties of the included domain [33].

### 2.2. IgG-like Bispecific Antibody Structures

IgG-like bsAbs maintain the basic antibody scaffold and contain an Fc region [4,5,37]. Compared with the fragment-based formats described above, these structures are known to extend serum half-life by FcRn-mediated recycling through the Fc region [4,37]. The inclusion of an Fc region that enables protein A-based purification has made bispecific antibodies with this structure advantageous in pharmaceutical process development [37,38]. Because of these properties, many bsAbs in current clinical trials have been developed from Fc-containing IgG-like structures [1,4,37]. IgG-like bsAbs are classified into symmetric and asymmetric formats according to whether overall structural symmetry is maintained [4,5,37].

#### 2.2.1. Symmetric Bispecific Antibodies

**Figure 2:**
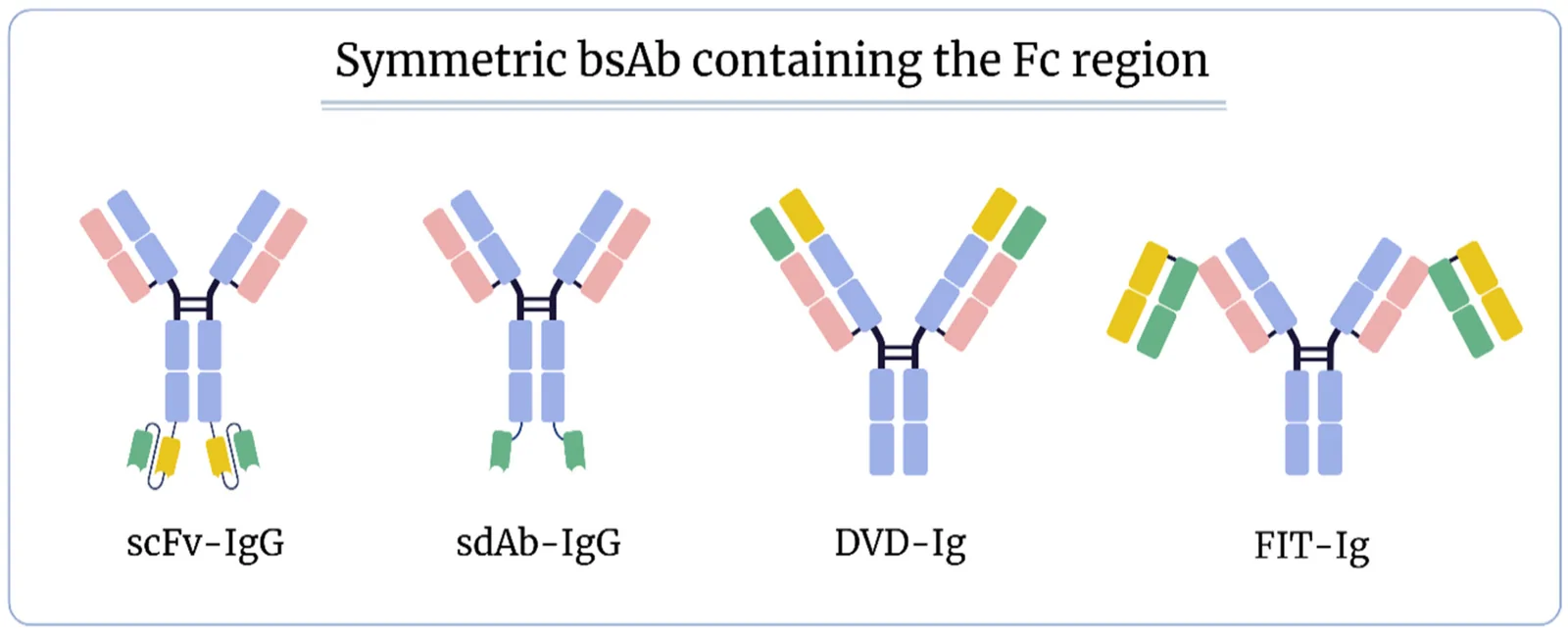
Symmetric IgG-like bispecific antibody architectures. Symmetric IgG-like bsAb formats retain the IgG scaffold while introducing additional antigen-binding modules or stacked variable domains. These formats include fusion-based, dual-variable domain, and tandem Fab designs. bsAb (Bispecific Antibody); scFv (Single-Chain Variable Fragment); sdAb (Single-Domain Antibodies); DVD-Ig (Dual-Variable-Domain Immunoglobulin); FIT-Ig (Fabs-in-Tandem Immunoglobulin).

Symmetric structures are formats that confer bispecificity while maintaining the intrinsic symmetry of IgG [4,5,37] (Figure 2). This structure is achieved by fusing additional antigen-binding modules to the Fab or Fc region or by stacking variable domains [4,5,15,16,39,40]. Because these structures are based on the repeated assembly of identical chains their manufacturing process is simpler than that of an asymmetric bispecific antibody, as it does not involve heavy-chain heterodimerization [4,5,37]. However, it is difficult to independently design the valency, relative positioning, and orientation of the binding modules for each antigen, thereby limiting independent control over the functional role of each antigen-binding module [4,5,7].

Fusion-based design. Fusion structures have gained bispecificity by fusing antigen-binding modules, such as scFv or sdAb, to the N- or C-terminus of a basic IgG scaffold. This design has the advantage of introducing additional binding modules while retaining the valency of the parental IgG [4,5,39]. However, Mingyan Cao et al. reported that engineered cysteine introduced to stabilize the additional scFv failed to form normal intramolecular disulfide bonds in some molecules. It induced abnormal intermolecular disulfide pairing, thereby forming dimer species. Therefore, in fusion structures, the folding stability of the added antigen-binding module and proper disulfide bond formation should be considered key design factors that affect production yield [39].

Dual-Variable-Domain Stacked Design. Dual-variable-domain formats are designed to recognize two targets simultaneously by stacking two variable domains in series on a single Fab arm. DVD-Ig (Dual-Variable-Domain Immunoglobulin), a representative format, acquires bispecificity by sequentially arranging an outer variable domain and an inner variable domain [15,16,40,41]. In this format, domain order and interdomain linker design directly affect antigen-binding site exposure and simultaneous binding potential [15,41]. Therefore, epitope location and structural compatibility must be considered during the design stage [7,15,41].

Tandem Fab-Based Design. Tandem Fab formats are designed to recognize two antigens by arranging Fab units in series on a single IgG arm [17,18]. For example, FIT-Ig (Fabs-in-Tandem Immunoglobulin) has achieved dual specificity by arranging Fab modules consecutively. Fab units already contain stabilized VH–VL and CH1–CL structures, providing an advantage in domain stability [17]. However, increasing molecular size and the appearance of spatial constraints due to additional Fab could lead to interference between binding modules. Therefore, it is important to balance between interdomain distance control and structural flexibility [17,18].

#### 2.2.2. Asymmetric Bispecific Antibodies

**Figure 3:**
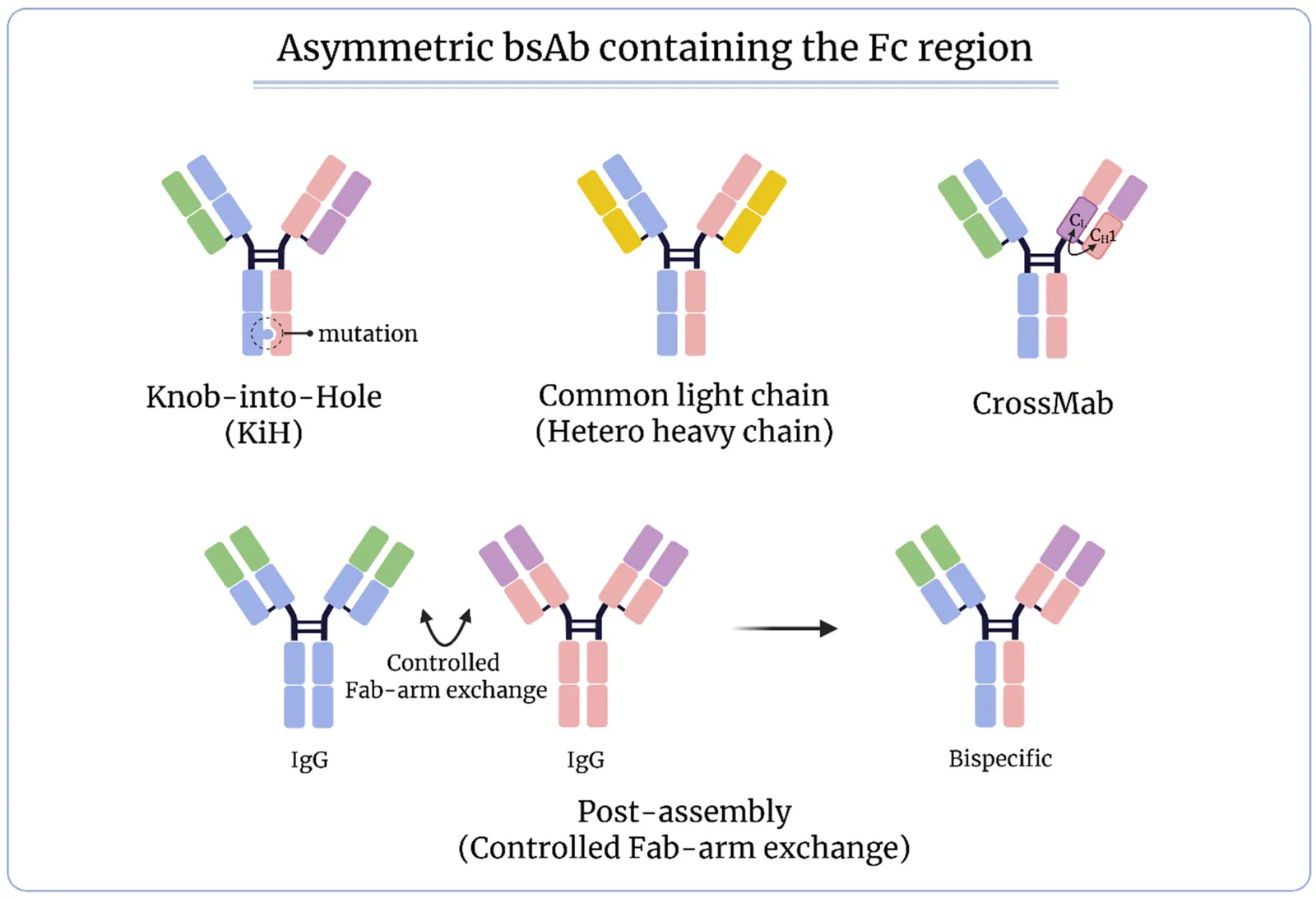
Asymmetric IgG-like bispecific antibody architectures. Asymmetric IgG-like bsAbs assign different antigen-binding specificities to separate Fab arms. Key design strategies include heavy chain heterodimerization, light chain-pairing control, and post-assembly Fab-arm exchange. bsAb (Bispecific Antibody); Fab (Fragment Antigen-Binding).

Asymmetric structures achieve bispecificity by assigning two different antigen-binding specificities to separate Fab arms within a single molecule [4,5,37] (Figure 3). Because each Fab arm is designed independently, these formats allow more precise control over antigen-specific binding affinity and functional roles [4,7,37]. Asymmetric formats require selective heterodimerization of two different heavy chains [19,20,21,42]. Light chain mispairing should also be prevented during intracellular assembly [22,23,24,42]. Thus, asymmetric structures offer greater design flexibility but also entail greater production and assembly complexity, thereby necessitating precise control of both heavy-chain dimerization and light-chain pairing [4,37,42].

Heavy Chain Heterodimerization-Based Design. Heavy chain heterodimerization-based design is a strategy used in asymmetric bsAbs to achieve bispecificity by promoting selective pairing between two different heavy chains. A representative approach is to engineer the CH3 domain interface to favor heterodimer formation over homodimer formation [19,20,21]. The Knob-into-Hole (KiH) strategy, developed by Ridgway, John BB. et al., is a well-known example. In this strategy, a bulky amino-acid substitution was introduced into one heavy chain to generate a “knob,” whereas a complementary cavity, or “hole,” was engineered into the other heavy chain. This design tended to suppress homodimer formation and favored the formation of the desired heterodimer [21]. Strategies based on electrostatic complementarity or interface engineering have also been reported to improve heterodimer selectivity [19,20,43]. These approaches preserve the half-life benefit of Fc-containing antibodies through FcRn-mediated recycling and allow different antigen specificities to be assigned to each Fab arm [4,37]. However, several reports have indicated that mutations introduced into the CH3 interface affect not only heterodimer formation efficiency but also molecular stability, assembly behavior, and the generation of residual homodimer byproducts [20,42,43]. Therefore, these results suggest careful consideration of expression optimization, selective chain pairing, efficiency, assembly fidelity, and physicochemical stability in heavy chain heterodimerization-based design [4,20,37,42].

Light Chain-Pairing Control Strategy. Light chain-pairing control is a design strategy used to form correct antigen-binding sites in asymmetric bsAbs [22,23,24,42]. This strategy promotes selective pairing of each heavy chain with its intended light chain [22,23,24]. In asymmetric bsAbs, unintended heavy–light chain combinations increase functional heterogeneity and reduce the proportion of the desired bispecific molecule, leading to lower yield and greater purification complexity [22,23,42]. Therefore, strategies for selective light chain pairing are essential for the effective production of asymmetric bsAbs [22,23,24,42,44].

One representative approach is the common light chain (CLC) strategy. This strategy structurally reduces the possibility of light chain mispairing by designing the two antigen-binding specificities to share the same light chain. It reduces molecular diversity during assembly and improves functional homogeneity. In the CLC strategy, the combination of variable domains must be optimized to maintain binding specificity and affinity for each antigen, even when the two heavy chains share a single light chain [44,45].

Another representative strategy is the CrossMab approach. This method promotes selective pairing between the intended heavy and light chains by exchanging the positions of the CH1 and CL domains. In this design, the mispairing of heavy and light chains was shown to be suppressed by exploiting structural complementarity [22,23]. Interface engineering strategies had also been reported to strengthen complementary interactions by redesigning amino-acid sequences at the heavy–light chain contact interface [24]. These complementary strategies need to be implemented in tandem with selective chain pairing and careful consideration of how domain rearrangement or interface mutations affect molecular stability, expression yield, quality attributes, and process consistency [22,23,24,42].

Post-assembly-based Fab-arm Exchange Strategy. Post-assembly-based Fab-arm exchange is an approach that generates asymmetric bsAbs by first producing two separate monospecific IgGs and then exchanging their Fab arms during a process step. This strategy induces the rearrangement of Fab arms between two IgG molecules into the desired combination, using controlled reduction and reoxidation. This approach shifts the burden of simultaneous four-chain assembly from the intracellular stage to a post-production step. It has the benefit of generating bispecific molecules with relatively high purity. It also offers advantages in manufacturing reproducibility and product homogeneity. In post-assembly-based Fab-arm exchange, the accuracy and efficiency of the Fab-arm exchange reaction, process condition optimization, and process control during scale-up are critical design elements [25,26].

## 3. Relationship Between Bispecific Antibody Structure and Function

The binding diversity and functional flexibility of bsAbs are expanded by various structural modifications. These variations have been reported to contribute to improved antigen-recognition precision and pharmacological performance [4,6,7]. Therefore, the correlation between the factors that determine the three-dimensional structure of bsAbs and the functions defined by those factors is considered a key design parameter governing the balance between the efficacy and safety of bsAbs [6,7,9].

### 3.1. Valency

Valency refers to the number of binding sites that a single bsAb molecule has for a given antigen [6,7,46]. Monovalent interaction is formed by a single available binding site for each antigen. Bivalent or multivalent interactions are enabled by the presence of two or more binding sites [47,48].

Multivalent binding enhances avidity by increasing overall binding stability, as antigen engagement is maintained by the remaining binding sites after a single site dissociates. Avidity refers to the overall binding strength generated by multivalent interactions. This effect becomes more pronounced as antigen density on the cell surface increases. Functional avidity is achieved only when antigen density, epitope accessibility, and molecular geometry permit simultaneous engagement or productive rebinding [48]. Increased avidity influences binding persistence and selectivity on target cells. In contrast, monovalent binding provides limited avidity effects, resulting in relatively weaker binding persistence [47,48]. Thus, valency primarily controls antigen-density-dependent binding stability rather than serving as an independent measure of therapeutic potency [47,48].

The objective of valency design is to assign binding sites according to the function required from each target, rather than to maximize the total number of binding sites [6,7]. Additional valency toward a disease-associated antigen can increase antigen-density-dependent avidity and stabilize cell-surface binding when the target distribution and molecular geometry permit multivalent engagement [48]. For an activating immune-cell receptor, the priority is to achieve sufficient but controlled effector-cell engagement, because changes in molecular format and valency can alter T-cell binding and functional activity [46]. In receptor-targeting bsAbs, multivalent or biparatopic engagement can instead be used to reorganize receptors; the resulting receptor clustering and signaling effects depend on epitope arrangement and binding-domain orientation [49,50]. Therefore, optimal valency is the target-specific distribution of binding sites which produces the intended biological output without unnecessary or nonproductive engagement (Table 2).

### 3.2. Epitope Geometry

Epitope geometry refers to the spatial arrangement formed when the two binding moieties of a bsAb engage their targets [7,8,54]. It includes the distance between two epitopes, their orientation on the cell surface, and their position relative to the cell membrane [7,55,56]. These factors determine the likelihood of simultaneous binding and the stability of the binding complex [7,8,54]. They also affect how a bsAb bridges and organizes targets on the cell surface [7,54,56].

The objective of epitope-geometry design is to generate the spatial arrangement required for the intended mechanism of action, rather than simply to minimize the distance between epitopes [7]. Simultaneous engagement is favored when the two epitopes are accessible and presented in orientations compatible with the molecular architecture of the bsAb [8]. Different target-bridging geometries can alter intercellular distance, receptor organization, and complex stability, thereby affecting synapse formation or receptor signaling [54].

Epitope geometry is particularly important in immune-cell-redirecting bsAbs because the position of the target epitope determines the spacing between the immune cell and the target cell. The relevant design goal is to select an epitope that supports close cell–cell contact and productive immune synapse formation. Membrane-proximal epitopes can be advantageous in this context, although antigen size and molecular format must also be considered [55]. This relationship was directly demonstrated for FcRH5 × CD3 bsAbs, in which the recognition of a membrane-proximal FcRH5 epitope promoted T-cell synapse formation and myeloma-cell killing [56].

For biparatopic bsAbs that recognize two epitopes on the same receptor, the design objective is to select an epitope pair that produces the receptor organization required for the intended function. Productive crosslinking of adjacent receptors can induce receptor clustering and signaling modulation, whereas dual-epitope occupancy without an appropriate spatial arrangement may not generate the same effect [49]. Biparatopic engagement can also enhance receptor internalization and downregulation when the molecular geometry supports receptor crosslinking [57].

For bsAbs targeting two different receptors, epitope geometry should permit simultaneous engagement of both targets or create the receptor proximity required for receptor downmodulation and combined signaling inhibition [58].

Thus, optimal epitope geometry is not defined by the shortest epitope distance. It is defined by the post-binding arrangement of cells or receptors required to produce the intended biological function (Table 3).

### 3.3. Affinity and Kinetics

Affinity refers to the binding strength between an antibody and an antigen and is generally expressed as the equilibrium dissociation constant (*K*_D_). Kinetics refers to the rate characteristics that govern complex formation and dissociation. It is described by the association rate constant (*k*_on_) and the dissociation rate constant (*k*_off_) [61,62].

(1) $$A+B\;\begin{array}{c} \overset{{\;k}_{on\;}}{\to }\\ \underset{{\;k}_{off}}{\leftarrow } \end{array}\;AB$$

(2) $$K_{D}=\frac{k_{off}}{k_{on}}$$

*A* and *B* represent the two interacting molecules, and *AB* represents the resulting complex [61,62]. Even with the same affinity, cellular responses can differ depending on how rapidly binding occurs and how long the complex is retained. In particular, the dissociation rate constant (*k*_off_) is directly related to binding residence time [9,62,63,64]. Thus, *k*_off_ affects the residence time on the target cell surface and the final functional output [9,63,64,65].

Identical affinity or kinetics are not required between the two binding arms of a bsAb. One binding arm is designed to maintain the stability of the overall complex. The other is assigned a role in regulating the directionality or dynamics of complex formation through transient association and dissociation [61]. Therefore, bsAb design requires tuning the K_D_, *k*_on_, and *k*_off_ of each binding arm according to the intended mechanism of action [9,52,53,63,64,65].

In T-cell-engaging bsAbs, the tumor-antigen-binding arm and the CD3-binding arm perform different functions. The tumor-binding arm should provide sufficient target cell retention while maintaining discrimination from normal cells with lower antigen expression. The CD3-binding arm should support productive T-cell recruitment without unnecessarily prolonging systemic T-cell activation. Therefore, tumor-arm affinity and CD3 affinity should not be optimized independently [9,52,64,65].

The functional effect of CD3 affinity also depends on molecular architecture. Tumor-antigen valency and affinity determine the stability of target-cell anchoring, whereas CD3 valency and binding-arm arrangement influence the number and persistence of T-cell interactions. Consequently, the same intrinsic CD3 affinity can produce different functional activity in different molecular formats [8,46,48].

The preferred affinity combination further depends on the therapeutic context. Abundant and accessible target-antigen expression may permit effective cytotoxicity with attenuated CD3 binding. In contrast, heterogeneous or low antigen expression may require stronger target-cell anchoring. However, increasing tumor-arm affinity can reduce discrimination from normal tissues, whereas increasing CD3 affinity can enhance T-cell activation and cytokine release syndrome (CRS). Excessive attenuation of CD3 affinity can also reduce cytotoxic activity. Affinity design therefore requires a balance among efficacy, CRS risk, and target specificity [52,64,65].

This trade-off has been demonstrated in T-cell-engaging bsAbs. The attenuation of CD3 affinity in a PSMA × CD3 bsAb reduced cytokine release while preserving target-dependent tumor-cell killing [52]. A CD19 × CD3 bsAb incorporating a low-affinity CD3-binding arm similarly maintained potent cytotoxicity with limited cytokine release in preclinical B-cell lymphoma models [66].

Affinity and kinetic asymmetry can also serve non-T-cell-engaging mechanisms. Emicizumab bridges FIXa and FX to replace the cofactor function of factor VIIIa [61,67]. Mak et al. reported K_D_ values of approximately 0.05 μM for FX and 5 μM for FIX, with corresponding dissociation half-lives of approximately 20 s and 1 s, respectively [61]. Under the reported assay conditions, the more stable FX interaction contributed to ternary-complex stability, whereas the more transient FIX interaction supported dynamic complex formation.

Thus, affinity and kinetics should be designed as coordinated properties of the two binding arms. Their optimal combination is determined by the intended mechanism of action, molecular architecture, target expression, and required therapeutic window rather than by the maximum achievable affinity (Table 4).

### 3.4. Linker

A linker refers to an amino-acid sequence that connects two domains or binding modules within a bsAb. Linkers are structural elements that control interdomain distance, relative orientation, and molecular flexibility [34,68]. The length and sequence properties of a linker affect the binding geometry that is formed with the two binding moieties. This effect leads to differences in simultaneous binding potential, binding stability, and functional output [34,69,70].

Linkers used in antibody engineering are generally classified as flexible linkers, which provide interdomain flexibility, and rigid linkers, which restrict relative orientation. Gly–Ser-based sequences are representative flexible linkers that permit interdomain movement and can reduce steric interference between adjacent domains. In contrast, rigid linkers restrict relative movement and maintain a more defined separation or orientation. Linker flexibility should therefore be selected according to the spatial requirements of the connected domains [34,68].

Linker length is another key variable that modulates interdomain distance and structural degrees of freedom. A linker that is too short can restrict the intended domain arrangement and increase steric interference. Conversely, excessive linker length can increase conformational heterogeneity and reduce the probability of maintaining a binding-competent domain arrangement [34,69,70].

The functional consequence of linker design depends on the molecular format. In single-chain fragment formats, linker length and sequence primarily regulate variable-domain pairing and oligomeric assembly. In fusion or stacked-domain formats, they primarily regulate binding-site accessibility and interdomain interference [15,69,70].

A single-chain diabody (scDb) is a representative example in which assembly behavior is determined by linker length. A scDb consists of four variable domains arranged in series on a single chain, with three linkers between them. In a VH–VL orientation, the arrangement is VH–linker–VL–middle linker–VH–linker–VL. Völkel et al. reported that a middle linker of at least 15 residues favored monomeric scDb formation, whereas shortening the middle linker to fewer than 13 residues increased dimer formation [69]. In DVD-Ig molecules, linker design altered the accessibility and association rate of the inner variable domain [15].

Therefore, linkers should be designed to support molecular assembly and antigen accessibility appropriate for each molecular format while minimizing structural interference and instability. The optimal linker sequence, length, and flexibility depend on the connected domains, intended assembly behavior, and molecular format (Table 5).

### 3.5. Fc-Region Design

The Fc (fragment crystallizable) region is the C-terminal constant-region portion of an IgG antibody and is composed of the lower hinge and paired CH2 and CH3 domains. Unlike the variable domains, the Fc region does not determine antigen specificity. Instead, it interacts with the neonatal Fc receptor (FcRn), Fcγ receptors (FcγRs), and complement component C1q, thereby controlling systemic persistence and immune effector activity [72,73,74].

FcRn binding protects internalized IgG from intracellular degradation and returns it to the circulation, thereby extending systemic exposure [73]. FcγR binding connects antigen-bound antibodies to immune effector cells, whereas the organization of Fc regions on an antigen-bearing surface can recruit C1q and initiate complement activation [72,74]. These interactions are influenced by the Fc amino-acid sequence, IgG subclass, glycosylation, and the spatial arrangement formed after antigen binding [72,74,75].

In Fc-containing bsAbs, the Fc region is not merely a scaffold or half-life-extension module. FcRn determines systemic exposure, whereas FcγR and C1q interactions can introduce immune-cell recruitment and complement activation in addition to the functions mediated by the two antigen-binding arms. In asymmetric IgG-like bsAbs, the CH3 interface also controls selective pairing of the two different heavy chains. Therefore, Fc design can affect pharmacokinetics, immune effector activity, and molecular assembly simultaneously [19,21,72,73,74].

The required Fc activity should be determined by the intended mechanism of action. When the therapeutic function should be mediated primarily through defined engagement of the two antigen-binding arms, additional FcγR-mediated cell recruitment or C1q-mediated complement activation may be unnecessary. In this context, effector-silent Fc variants can be used to reduce FcγR and C1q interactions while preserving FcRn binding and Fc stability [76].

Conversely, Fc function can be retained or enhanced when the recruitment of innate immune cells or complement activation contributes to the intended therapeutic mechanism. FcγR binding can be modified through Fc amino-acid substitutions or glycoengineering. In particular, reduction in core fucosylation increases FcγRIIIa binding and antibody-dependent cellular cytotoxicity without necessarily increasing FcRn or C1q binding [72,75]. Complement activation can also be regulated by Fc–Fc interactions because antigen-bound IgG molecules form ordered Fc assemblies that promote C1q recruitment [74].

Thus, Fc-region design in bsAbs should independently consider three properties: FcRn-mediated systemic exposure, FcγR/C1q-mediated effector activity, and CH3-dependent heavy-chain assembly. The optimal Fc configuration should preserve, attenuate, or enhance these properties according to the functional output required from the bsAb (Table 6).

## 4. Preclinical and Clinical Comparison of bsAb Structures Within the Same Target Combination

In preclinical and clinical applications of bsAbs, even though they have the same antigen combination, the antitumor efficacy, pharmacokinetic properties, administration methods, and toxicity management strategies are altered by the structural format [2,3]. Affinity asymmetry between the tumor-antigen-binding arm and the CD3-binding arm has also been used to modulate the balance between target-cell recognition and T-cell activation [3,66].

The comparisons below focus on how structural variables including Fc inclusion, valency, affinity distribution, epitope geometry, and Fc activity may contribute to functional and therapeutic differences within the same target combination. However, clinical outcomes cannot be attributed to molecular architecture alone because disease subtype, patient population, treatment line, dose, administration route, step-up dosing, premedication, and trial phase also affect efficacy and safety. Therefore, these comparisons are intended to identify structure–function relationships and therapeutic trade-offs rather than to establish direct clinical superiority among molecules evaluated in different studies.

### 4.1. CD19 × CD3

**Figure 4:**
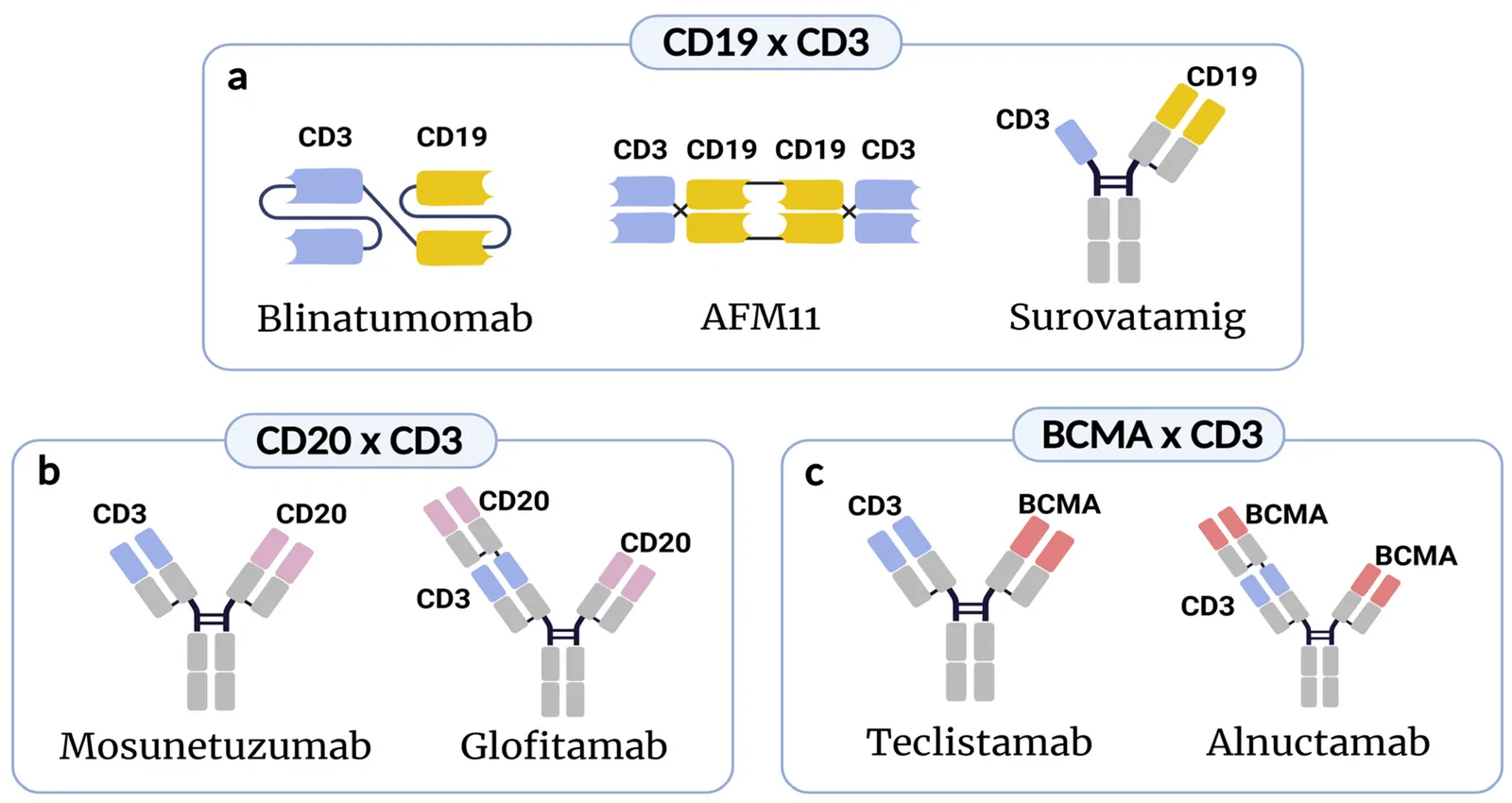
Structural comparison of T-cell-redirecting bispecific antibodies targeting CD19 × CD3, CD20 × CD3, and BCMA × CD3. (**a**) CD19 × CD3 bsAbs, including blinatumomab, AFM11, and surovatamig, represent fragment-based, tetravalent TandAb, and Fc-containing IgG-like formats. (**b**) CD20 × CD3 bsAbs, including mosunetuzumab and glofitamab, illustrate 1 + 1 and 2 + 1 IgG-like architectures. (**c**) BCMA × CD3 bsAbs, including teclistamab and alnuctamab, illustrate 1 + 1 and 2 + 1 IgG-like architectures. bsAb (bispecific antibody); BCMA (B-cell maturation antigen).

For bsAbs targeting CD19 × CD3, a wide range of formats has been developed, from fragment-based structures to 1 + 1 IgG-like structures (Figure 4a). The central structural question in this target combination is how CD19 and CD3 valency, intrinsic CD3 affinity, and Fc-mediated exposure should be combined to achieve stable tumor-cell engagement without excessive or prolonged T-cell activation.

#### 4.1.1. Fragment-Based Structures

The most representative example is blinatumomab. Blinatumomab is a BiTE tandem scFv molecule composed of two scFvs linked in series, which recognize CD19 and CD3, respectively, and form a 1 + 1 fragment-based structure without an Fc region. In the phase 3 TOWER study, overall survival was significantly expanded by blinatumomab, and a higher complete remission rate was achieved within 12 weeks than with standard chemotherapy in patients with relapsed or refractory B-ALL. Specifically, median overall survival was 7.7 months versus 4.0 months, and completed remission with full, partial, or incomplete hematologic recovery reached 44% versus 25%. However, continuous administration of blinatumomab was required because of its short half-life, reflecting a key structural limitation of fragment-based engagers [2,3,77].

Blinatumomab demonstrates that monovalent CD19 and CD3 binding can provide sufficient target-cell bridging when intrinsic affinity and epitope geometry support productive immune synapse formation [7,8,9].

Meanwhile, AFM11 features enhanced multivalency [30,78]. It adopts a tetravalent TandAb format with two binding sites each for CD19 and CD3. With increased valency, AFM11 demonstrated enhanced cytotoxic activity in preclinical studies [30]. However, in a phase 1 clinical study for patients with NHL, neurological dose-limiting toxicity occurred in 5 of 16 patients, corresponding to 31.3%. In addition, no objective response was observed in the NHL cohort and was limited in the ALL cohort. As a result of this trial, further development was ultimately discontinued [78].

Compared with blinatumomab, AFM11 changes not only total valency but also the distribution of avidity across both sides of the immune synapse. Bivalent CD19 engagement can stabilize tumor-cell anchoring, whereas bivalent CD3 engagement can increase the number and persistence of T-cell interactions. The 2 + 2 format can therefore strengthen productive engagement but may also increase the duration or intensity of T-cell engagement [8,9,30,46,48].

The available studies do not demonstrate that the 2 + 2 structure directly caused the neurological toxicity or limited clinical activity of AFM11 because affinity, dose, exposure, disease, and administration schedule were not controlled in a matched comparison. AFM11 shows that increased valency and preclinical cytotoxicity do not necessarily result in a wider clinical therapeutic window [8,9,30]. The functional consequence of valency depends on which target receives the additional binding sites and whether the resulting avidity supports the intended mechanism [46,48,78].

#### 4.1.2. Asymmetric IgG-like Structures

Surovatamig was developed as an Fc-containing CD19 × CD3 bsAb with attenuated CD3 binding [66,79]. Preclinical studies showed that surovatamig maintained CD19-dependent cytotoxicity while producing less cytokine release than a comparator with stronger CD3 engagement [66]. Its Fc-containing format supports prolonged systemic exposure and intermittent administration, whereas the available clinical data provide early evidence of activity rather than a matched demonstration of reduced immune-mediated toxicity [66,79].

Unlike the 2 + 2 AFM11 format, surovatamig retains monovalent CD3 engagement and combines sustained CD19-dependent target-cell recognition with attenuated intrinsic CD3 affinity. The Fc-containing architecture prolongs systemic exposure, whereas the attenuated CD3-binding arm regulates the strength of each individual T-cell interaction. This design therefore separates exposure duration from the intensity of CD3-mediated engagement rather than increasing avidity on both sides of the immune synapse [53,63,64,65,66].

The functional effect of CD3 affinity must consequently be interpreted together with molecular format and systemic persistence. In a long-lived Fc-containing molecule, excessively strong CD3 binding may prolong systemic T-cell activation, whereas excessive affinity attenuation may compromise cytotoxic activity. Surovatamig therefore illustrates the need to coordinate Fc-mediated exposure and intrinsic CD3 affinity rather than maximizing either property independently [63,64,65,66].

#### 4.1.3. Structure–Function Implications

The CD19 × CD3 examples demonstrate that valency, intrinsic CD3 affinity, and Fc-mediated exposure perform complementary functions. Blinatumomab uses monovalent engagement with short systemic persistence, AFM11 distributes avidity to both the tumor-cell and T-cell sides, and surovatamig combines prolonged exposure with attenuated CD3 affinity [27,30,66]. Accordingly, CD19 × CD3 design cannot be ranked simply as 1 + 1 versus 2 + 2. The relevant comparison is how each architecture distributes target-cell retention, CD3 engagement, and exposure duration. Additional valency can be advantageous when it produces productive target-dependent avidity, whereas attenuated CD3 affinity can regulate T-cell activation when sufficient tumor-cell anchoring is maintained [63,64,65,66].

### 4.2. CD20 × CD3

For bsAbs targeting CD20 × CD3, both 1 + 1 and 2 + 1 IgG-like formats have entered clinical development (Figure 4b). The principal structural comparison is whether CD20-dependent target-cell retention is mediated only by monovalent intrinsic binding properties or is additionally supported by a second CD20-binding site, while CD3 engagement remains monovalent [8,9,80,81].

#### 4.2.1. 1 + 1 Asymmetric IgG-like Structures

Mosunetuzumab has a CD20 × CD3 1 + 1 asymmetric IgG-like structure [82,83]. In a multicenter phase 2 study of patients with relapsed or refractory follicular lymphoma, a CR rate of 60.0% was reported. CRS occurred in 44% of patients, with most events being grade 1–2 and occurring mainly during the first cycle [82]. Step-up dosing was used to manage early cytokine release, whereas the Fc-containing IgG-like format enabled intermittent systemic administration [82,83].

Another example of a similar 1 + 1 IgG-like structure is epcoritamab [84,85]. In the initial phase 1/2 study, the diffuse large B-cell lymphoma cohort showed clinical responses, and most CRS events were grade 1–2 [84]. In the subsequent long-term follow-up of the pivotal EPCORE NHL-1 study, patients with relapsed or refractory large B-cell lymphoma achieved an ORR of 63.1% and a CR of 40.1% [85].

Mosunetuzumab and epcoritamab demonstrate that a 1 + 1 arrangement can provide clinically effective CD20-dependent T-cell redirection without target-side bivalency. In this architecture, ternary-complex stability depends more directly on CD20 density, intrinsic arm affinity and kinetics, epitope accessibility, and systemic exposure [8,9,46,48].

Because the two molecules share the same broad valency arrangement but were evaluated in different lymphoma populations using different routes and dosing schedules, they are not a structural comparison with each other. Rather, together, they establish that a 1 + 1 format can support effective CD20-dependent T-cell redirection in different clinical settings [82,83,84,85].

#### 4.2.2. 2 + 1 Asymmetric IgG-like Structures

Glofitamab has a 2 + 1 structure that binds bivalently to CD20 and monovalently to CD3. This structure was designed to increase CD20-dependent avidity while avoiding the additional avidity that would arise from bivalent CD3 engagement [80,81]. In a pivotal phase 2 study of patients with relapsed or refractory DLBCL, it was reported that an ORR of 52% and a CR rate of 39% were achieved with glofitamab. CRS was mainly grade 1–2, and grade 3 CRS was observed in 3–4% of patients [86].

Glofitamab redistributes valency toward the tumor antigen. The second CD20-binding site can increase antigen-density-dependent avidity and rebinding on CD20-positive cells, whereas monovalent CD3 engagement avoids the additional avidity that would result from bivalent CD3 engagement. The 2 + 1 architecture therefore seeks to strengthen tumor-cell anchoring without proportionally increasing CD3 valency [46,48,80,81].

CD3 monovalency should not be interpreted as equivalent to attenuated intrinsic CD3 affinity. Valency determines the number of possible CD3 interactions, whereas affinity and kinetics determine the strength and duration of each interaction. A monovalent CD3 arm can therefore still induce strong T-cell activation when its intrinsic affinity, epitope geometry, and systemic exposure are sufficient [52,53].

#### 4.2.3. Structure–Function Implications

The CD20 × CD3 comparison illustrates target-specific valency allocation. The 1 + 1 format relies on intrinsic affinity, antigen density, geometry, and systemic exposure to maintain tumor-cell engagement, whereas the 2 + 1 format introduces CD20-dependent avidity while retaining monovalent CD3 binding. The difference between these formats is therefore not a simple hierarchy of lower and higher total valency. It is a design choice regarding whether additional binding stability should be assigned specifically to CD20. This target-side avidity may strengthen tumor-cell retention without requiring a parallel increase in CD3 valency [46,48,80,81].

### 4.3. BCMA × CD3

bsAbs targeting BCMA × CD3 are among the most actively developed T-cell-redirecting bsAbs in multiple myeloma. For this target antigen combination, both 1 + 1 and 2 + 1 IgG-like structures were developed (Figure 4c). The central structural comparison is how binding stability is distributed between the myeloma-cell side and the T-cell side. The 1 + 1 formats use monovalent engagement of both BCMA and CD3, whereas alnuctamab combines BCMA bivalency with relatively low-affinity monovalent CD3 binding [52,53,87].

#### 4.3.1. 1 + 1 Asymmetric IgG-like Structures

Teclistamab is a representative example of a BCMA × CD3 1 + 1 IgG-like structure. In a phase 1/2 study, teclistamab showed an ORR of 63.0% and a CR or better rate of 39.4% in patients with triple-class exposed relapsed/refractory multiple myeloma. CRS occurred in 72.1% of patients, but grade 3 CRS occurred in only 0.6%, with most CRS events being grade 1–2 [88].

Another example with a similar 1 + 1 structure is elranatamab [89,90]. In a phase 2 study, elranatamab showed an ORR of 61.0% and a CR or better rate of 35.0%, while CRS occurred in 57.7% of patients and no grade 3–4 CRS was reported. The dosing schedule of the 50 patients who responded was switched to every 2 weeks. Forty of these patients, corresponding to 80.0%, maintained or improved their response for at least 6 months [89].

Teclistamab and elranatamab establish that intramolecular BCMA avidity is not required for clinically meaningful activity. In these 1 + 1 formats, productive immune synapse formation depends on BCMA availability, the intrinsic affinity and kinetics of each arm, epitope geometry, and systemic exposure [88,89,90].

#### 4.3.2. 2 + 1 Asymmetric IgG-like Structures

Alnuctamab is a representative example of a 2 + 1 IgG-like structure that binds bivalently to BCMA and monovalently to CD3. Alnuctamab uses a different binding-control strategy. Bivalent BCMA engagement allows avidity to form when both BCMA-binding sites can productively engage the myeloma-cell surface, whereas the relatively low-affinity CD3 arm limits the strength of each individual T-cell interaction. The reported CD3-binding K_D_ of approximately 70–100 nM supports this asymmetric distribution of binding strength [52,53,87].

In the phase 1 study, the ORR was 58.9% among all subcutaneously treated patients and 71.4% in the 30 mg cohort. CRS occurred in 57.9% of subcutaneously treated patients, with no grade 3–4 CRS. Subcutaneous administration was selected for further evaluation after showing a more favorable tolerability profile than intravenous administration [87].

The 2 + 1 architecture should therefore be interpreted as a mechanism for separating tumor-cell anchoring from T-cell triggering. BCMA bivalency shifts binding stability toward the target-cell side, while attenuated monovalent CD3 binding regulates the activation signal delivered through the immune synapse. This differs from the 1 + 1 strategy, in which target-cell anchoring and T-cell engagement both depend primarily on single-arm binding properties [8,9,87].

#### 4.3.3. Structure–Function Implications

The BCMA × CD3 comparison demonstrates a coordinated valency–affinity strategy. The 1 + 1 molecules rely on monovalent intrinsic binding properties and systemic exposure, whereas alnuctamab uses BCMA-dependent avidity to strengthen target-cell retention while permitting the attenuation of intrinsic CD3 affinity. Additional tumor-side valency can therefore stabilize target engagement without requiring a parallel increase in CD3 valency or CD3-binding strength [87,88,89,90].

### 4.4. HER2 × HER2

**Figure 5:**
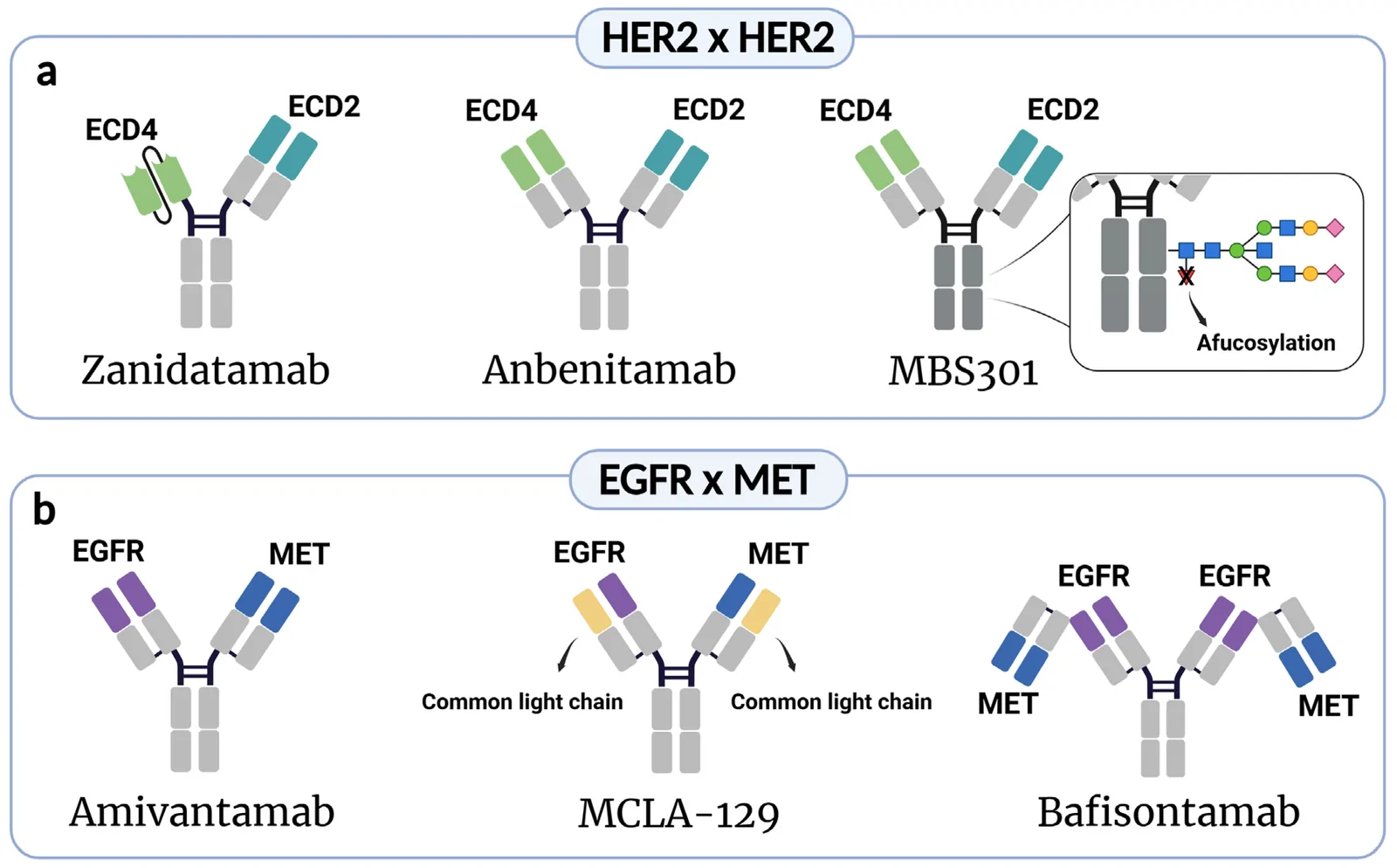
Structure-dependent mechanisms of biparatopic and dual-receptor bispecific antibodies targeting HER2 × HER2 and EGFR × MET. (**a**) HER2 × HER2 biparatopic bsAbs, including zanidatamab, anbenitamab, and MBS301, recognize two distinct HER2 epitopes, such as ECD2 and ECD4, and can induce receptor clustering, internalization, signaling modulation, and Fc-mediated effector functions. (**b**) EGFR × MET dual-receptor bsAbs, including amivantamab, MCLA-129, and bafisontamab, target EGFR and MET through distinct molecular architectures and can promote signaling blockade, receptor endocytosis, co-degradation, and antitumor activity. HER2 (Human Epidermal Growth Factor Receptor 2); EGFR (Epidermal Growth Factor Receptor).

Antibodies targeting HER2 × HER2 differ from typical bsAbs that simultaneously recognize two distinct antigens. Instead, they have been developed as biparatopic bsAbs that simultaneously recognize two different epitopes on the same receptor (Figure 5a). Because the molecules in this group recognize a similar HER2 epitope pair, the most informative comparison is not simply the presence of biparatopic binding. It is how Fab-mediated receptor organization and Fc-mediated immune-effector activity are combined within that biparatopic framework. Epitope geometry controls the arrangement, clustering, and trafficking of HER2 after binding, whereas Fc sequence and glycosylation control FcγR- and C1q-dependent functions [7,49,91].

#### 4.4.1. Asymmetric Biparatopic IgG-like Structures

A representative clinical example is zanidatamab [92]. Zanidatamab has a biparatopic IgG1-like structure that simultaneously recognizes extracellular domain II (ECD2) and extracellular domain IV (ECD4) of HER2. This structure enables dual-epitope engagement distinct from that of single HER2-targeting antibodies [49]. In the HERIZON-BTC-01 study of patients with HER2-positive biliary tract cancer, a confirmed ORR of 41.3%, a DCR of 68.8%, and a median DoR of 12.9 months were reported. These results demonstrated that a biparatopic structure exhibited clinically significant antitumor activity [92].

Mechanistic studies showed that ECD2/ECD4 engagement by zanidatamab can generate HER2 clustering, receptor internalization, signaling suppression, ADCC, ADCP, and complement-dependent cytotoxicity [49]. Its activity therefore reflects a coordinated mechanism in which biparatopic geometry reorganizes HER2 while the Fc region contributes immune-effector activity [49,92].

There is another biparatopic IgG1-like structure called anbenitamab (KN026). Anbenitamab simultaneously targets HER2 ECD4 and ECD2, corresponding to the trastuzumab- and pertuzumab-binding domains, respectively. In a first-in-human phase 1 study, an ORR of 28.1% and a median PFS of 6.8 months were achieved among 57 patients with HER2-positive metastatic breast cancer [93].

Anbenitamab provides an independent clinical example of the same broad ECD2/ECD4 targeting strategy. However, the available studies do not define whether its arm orientation and post-binding receptor organization reproduce those reported for zanidatamab. It therefore supports the therapeutic relevance of the epitope pair but does not by itself establish functional equivalence between the two biparatopic architectures [49,92,93].

Quantitative differences according to structural design were also identified at the preclinical level. MBS301 was developed as a HER2-targeting biparatopic bsAb with an afucosylated Fc. K_D_ values of 1.17 × 10^−7^ M and 2.35 × 10^−7^ M were measured for FcγRIIIa V158 and F158, respectively. Higher FcγRIIIa binding affinity than comparator antibodies was demonstrated by these values. In ADCC assays, EC50 values of 2.0 × 10^−3^ μg/mL and 0.2 × 10^−3^ μg/mL were obtained against SW480 and SK-OV-3 cell lines, respectively. Stronger cytotoxic activity was detected with MBS301 than with trastuzumab, pertuzumab, or their combination. In xenograft models, tumor inhibition rates of 97.18% in BT474 by day 28 and 100% in NCI-N87 were achieved [91].

MBS301 makes Fc enhancement an explicit structural intervention within a biparatopic HER2 antibody. Afucosylation increases FcγRIIIa binding and ADCC without changing which HER2 epitopes are recognized. Its design therefore demonstrates that Fc-mediated cytotoxicity can be strengthened independently of the Fab-level epitope combination [91].

#### 4.4.2. Structure–Function Implications

The HER2 × HER2 examples separate two functional layers of bsAb design. Zanidatamab provides mechanistic evidence that epitope geometry can reorganize HER2 and promote internalization and signaling modulation. MBS301 demonstrates that the immune-effector output of a biparatopic molecule can be increased through Fc glycoengineering. Anbenitamab confirms that ECD2/ECD4 recognition is clinically applicable but currently provides less mechanistic information regarding post-binding receptor organization. Accordingly, Fab geometry and Fc engineering are complementary but non-interchangeable design variables. Geometry determines what receptor arrangement is produced after biparatopic binding, whereas Fc engineering determines how strongly immune cells respond to the resulting antibody-coated target. A molecule optimized only for dual-epitope occupancy may not reproduce the receptor clustering of another biparatopic format, and a molecule optimized for clustering may still be further modified through Fc engineering to alter its effector profile [7,49,91,92,93].

### 4.5. EGFR × MET

bsAbs targeting EGFR × MET have been developed as a strategy to suppress resistance and signaling bypass by simultaneously blocking receptor tyrosine kinases with complementary roles in tumor cells (Figure 5b). The principal structural comparison is between 1 + 1 formats that engage EGFR and MET through one binding site per receptor and a tetravalent format capable of multivalent receptor crosslinking. The 1 + 1 formats can support dual signaling inhibition and receptor downmodulation, whereas tetravalent engagement can additionally promote higher-order receptor organization, internalization, and co-degradation [58,60,94,95,96].

#### 4.5.1. 1 + 1 Asymmetric IgG-like Structures

Amivantamab is a representative clinical example. Amivantamab is a fully human 1 + 1 IgG-like bsAb that simultaneously targets EGFR and MET. Clinical efficacy was confirmed in patients with EGFR exon 20 insertion NSCLC [58,60,97,98]. In the CHRYSALIS study, an ORR of 40% was reported among patients with disease progression after platinum-based therapy [97]. In the subsequent PAPILLON study conducted in the first-line setting, an ORR of 73% and a median PFS of 11.4 months were achieved with the amivantamab–chemotherapy combination. A significant improvement over chemotherapy alone was demonstrated [98].

Amivantamab assigns one binding arm to EGFR and one to MET. This arrangement supports simultaneous inhibition of both receptors and also promotes receptor downmodulation, degradation, trogocytosis, and Fc-dependent immune-cell recruitment [58,60]. Thus, the 1 + 1 format can combine dual-receptor blockade with receptor removal and Fc-mediated activity without receptor-specific bivalency.

MCLA-129 is another EGFR × MET-targeting bsAb with a 1 + 1 IgG-like structure. It was designed using a common light-chain-based platform to ensure uniformity of molecular assembly and developability [94,99]. In early clinical data, ORRs of 43.5%, 28.6%, and 21.8% were observed in cohorts with MET exon 14 skipping, EGFR exon 20 insertion, and sensitizing EGFR mutations, respectively [99]. In preclinical studies, MCLA-129 inhibited EGFR- and MET ligand-induced signaling. Dose-dependent tumor inhibition was also demonstrated in a patient-derived EGFR exon 20 insertion xenograft model [94].

MCLA-129 similarly uses 1 + 1 EGFR × MET engagement but incorporates a common-light-chain strategy to improve chain pairing and product homogeneity. This assembly strategy does not itself determine receptor signaling; the functional effects are mediated by the EGFR/MET-binding arms and Fc properties [44,45,94].

#### 4.5.2. Symmetric Tetravalent IgG-like Structures

Bafisontamab (EMB-01) was developed as a tetravalent EGFR × MET bsAb [95,96]. In an early clinical study, 2 partial responses and 14 cases of stable disease were reported among 38 response-evaluable patients with NSCLC [100]. In a recent mechanistic study, bispecific binding was maintained by bafisontamab. EGFR/c-Met endocytosis and co-degradation were also promoted. Stronger inhibition of downstream signaling was demonstrated than with parental mAb monotherapy or combination therapy [96].

Unlike the 1 + 1 formats, bafisontamab provides additional EGFR- and MET-binding sites that can increase receptor occupancy and promote multivalent receptor crosslinking. This higher-order engagement is associated with receptor internalization and EGFR/MET co-degradation, extending the mechanism beyond simultaneous signaling blockade [7,46,48,95,96].

#### 4.5.3. Structure–Function Implications

The principal difference between these formats lies in the receptor-level mechanism generated after binding. The 1 + 1 formats provide simultaneous EGFR and MET recognition and can combine signaling inhibition, receptor downmodulation, and Fc-mediated immune activity. The tetravalent format adds multivalent receptor organization that can promote internalization and co-degradation of both receptors. Thus, valency does not simply increase binding strength in EGFR × MET bsAbs. When combined with an appropriate arm arrangement and epitope geometry, additional valency can change the mechanism from dual-receptor blockade toward coordinated receptor trafficking and degradation [94,95,96].

## 5. In Silico-Guided Optimization of Structure–Function Determinants

**Figure 6:**
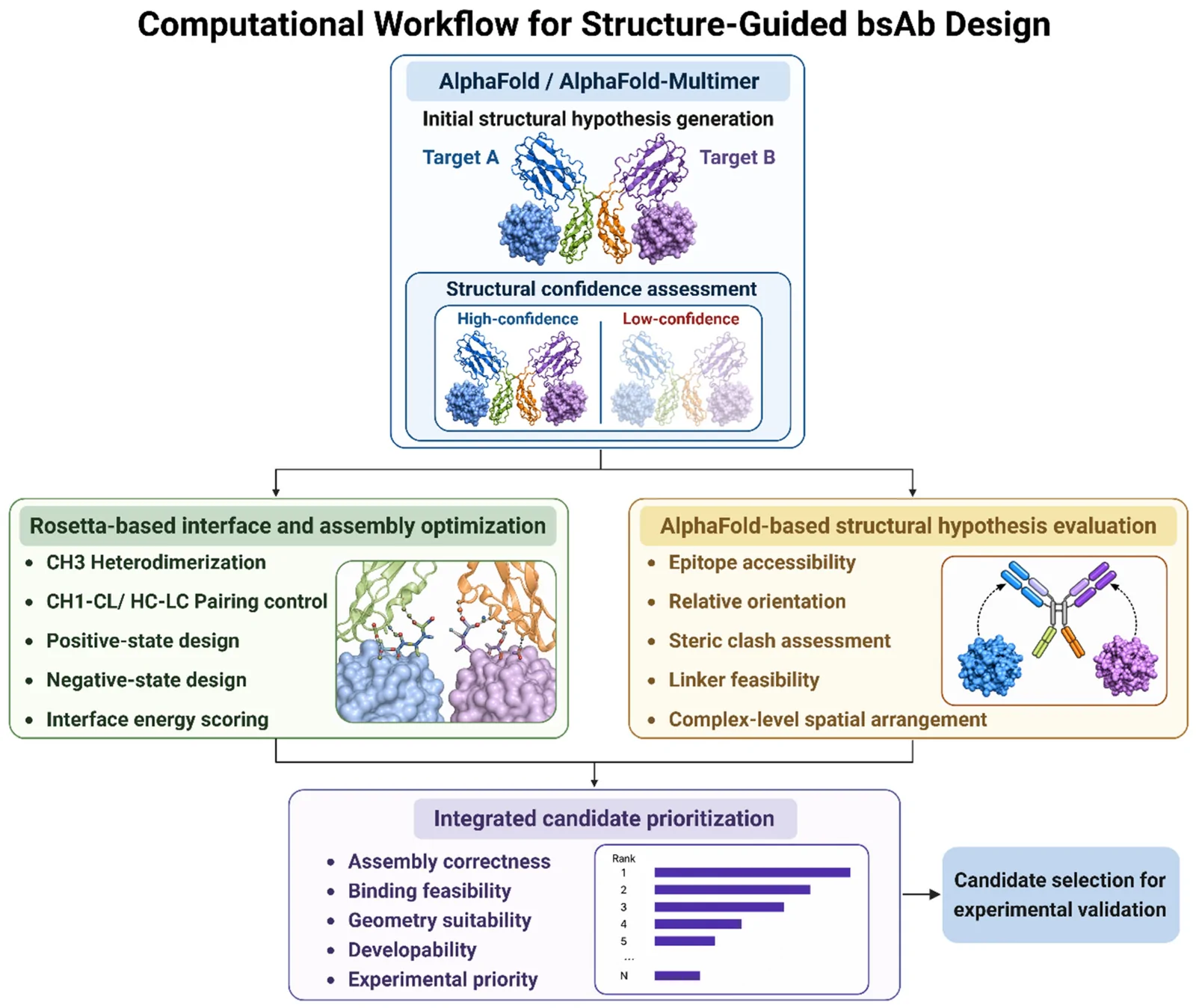
In silico-guided optimization workflow for bispecific antibody structure–function design. Structure-guided computational approaches can support rational bsAb design by linking molecular architecture to functional output. Rosetta-based modeling is suited for interface and assembly optimization, including CH3–CH3 heterodimerization, CH1–CL light chain pairing, interface-energy evaluation, and multistate design. Within this framework, positive-state design favors the intended assembly, whereas negative-state design disfavors competing homodimers or incorrect chain pairings. AlphaFold-based modeling is suited for structural hypothesis generation, including prediction of binding arm orientation, epitope geometry, steric compatibility, and potential receptor engagement modes. These tools can be integrated with linker modeling, affinity and kinetic design, and developability assessment to prioritize candidates before experimental validation. This workflow supports the transition from empirical format selection to predictive structure–function design in bsAb development. bsAb (bispecific antibody).

The function of a bsAb is not explained only by dual-target recognition. Functional output from structural elements, such as valency, epitope geometry, affinity, kinetics, and linker design, should be considered [4,6,7]. Therefore, recent bsAb development has expanded beyond empirical screening to include design and candidate selection using structure-based computational models (Table 7). In this process, Rosetta and AlphaFold serve complementary purposes in bsAb optimization [10,11,12,13] (Figure 6).

### 5.1. Rosetta-Based Interface and Assembly Optimization

Rosetta is an energy-based design tool that operates based on protein structures and interfaces [10]. In bsAb design, Rosetta is suitable for optimizing the assembly and stability of predefined structural interfaces [10,43,101]. It is particularly useful for multistate design and negative-state design strategies. In multistate design, candidate sequences are evaluated across multiple relevant structural states, including the intended complex and competing assembly states, so that mutations can be selected according to their relative compatibility with these states. Negative-state design explicitly includes undesired assemblies, such as heavy-chain homodimers or incorrect chain pairings, and seeks mutations that destabilize these negative states while preserving the intended heterodimeric interface [43,101]. In the context of bsAbs, these approaches enable the desired chain-pairing state to be optimized relative to competing products rather than evaluating the intended interface in isolation [43,101].

These characteristics are useful for addressing assembly challenges in bsAbs [10,42,43,101]. Correct chain pairing and stable assembly are critical developability challenges in bsAb [4,24,42]. In a study by Lewis et al., the CH1–CL interface was redesigned using structure-based computational modeling to generate orthogonal Fab interfaces that favored the intended heavy–light chain pairing [101]. Leaver-Fay et al. also reported a bispecific Fc with an optimized CH3 heterodimer interface using Rosetta-based negative-state repertoire design. In this approach, alternative homodimer conformations were treated as competing negative states during sequence selection, enabling the desired heterodimer to be favored relative to multiple undesired assemblies [43].

Thus, Rosetta is utilized for selecting and optimizing promising candidates based on bsAb assembly and interface quality. This approach is important for reducing chain-pairing errors and assembly instability at the design stage. It also supports rational molecular design in which the intended interface, competing assembly states, and developability-related constraints are considered before experimental candidate selection [4,10,42,43,101].

### 5.2. AlphaFold-Based Structural Hypothesis Generation

AlphaFold is a structure prediction tool used to predict protein and protein-complex structures and to evaluate relative molecular arrangement and structural compatibility [11,12,13]. This tool is suitable for examining the relative orientation of two binding moieties, epitope accessibility, potential structural interference, and complex-level structural arrangement [7,11,12,13,102,103]. AlphaFold-Multimer was introduced as an extended model for protein complex prediction [12]. AlphaFold 3 further expanded this predictive capability to biomolecular interactions involving proteins and other molecular components [13].

These features are important during structural hypothesis generation in bsAb design. In bsAbs, the final functional output is dependent on the relative arrangement of the two binding moieties. Therefore, the relative positioning of the binding moieties and the spatial compatibility of the target epitopes are important structural considerations [7,8,55,56]. AlphaFold-based models can therefore be used to examine whether simultaneous target engagement is structurally plausible and to identify potential steric interference between binding domains. However, the predicted structure alone does not determine binding affinity, binding kinetics, or biological activity. In a study by Zhang et al., AlphaFold-Multimer was used to predict the structure of a biparatopic antibody–target complex and to select candidates with structurally compatible binding arrangements [102]. Driscoll et al. also used AlphaFold-Multimer to predict complexes between the HER2 extracellular domain and nanoHER2 binders and examined the predicted binding interface and contributions of specific loop regions [103]. These examples indicate that AlphaFold-based modeling can support structural-compatibility assessment, binding-interface analysis, and candidate prioritization in biparatopic or dual-targeting antibodies [102,103].

Thus, AlphaFold is useful for predicting the overall structural arrangement and epitope geometry of bsAbs. This approach allows structurally plausible candidates to be selected and potential steric interference to be assessed in advance. Therefore, AlphaFold is suitable for structural hypothesis generation and candidate screening at the early stage of bsAb design [11,12,13,102,103].

## 6. Conclusions

bsAb structure design is as important as target selection because it determines whether the intended mechanism of action or functional enhancement is achieved. Through comparisons among the cases discussed in this review, it was summarized that, even with the same target combination, efficacy, toxicity, and pharmacokinetic properties vary depending on valency, epitope geometry, binding affinity and kinetics, linker architecture, and Fc-region design [4,6,7].

bsAb design should be directed toward implementing the functional outputs required for each therapeutic mechanism. In addition, the balance between efficacy and safety must be considered in bsAb design. Valency and CD3 affinity can increase the risks of excessive immune activation and cytokine release, whereas complex molecular architectures and inappropriate domain arrangements can introduce steric hindrance and developability limitations. Controlling toxicity and production-related limitations while maintaining the functionality required for the intended therapeutic mechanism is a major challenge.

AI-driven strategies could become increasingly important for rapidly and precisely exploring these structure–function balance points. Rosetta and AlphaFold can support interface and assembly optimization and structural hypothesis generation, respectively; however, these tools should be used to prioritize candidates rather than replace biochemical, biophysical, and functional validation. The competitiveness of bsAbs would be determined less by structural diversity itself than by the ability to interpret and integrate structure–function relationships into rational molecular design. While cases selected within the same structural classification and antigen combination were compared in this review, there are limitations: heterogeneity between studies, differences in evaluation metrics and analysis criteria, differences in preclinical models and clinical designs, and data maturity were not sufficiently controlled. To address these limitations and advance AI and machine learning-based structure–function analysis, it is necessary to establish a standardized archive that integrates preclinical and clinical results for bsAbs of various structures.

## Figures and Tables

**Table 1:** Structural characteristics of bsAb formats.

Structure	Subcategory	Representative Formats	Structural Characteristics	Functional Implications	Development and Manufacturing Considerations	Reference
Fragment-based	-	Tandem scFv, diabody, DART, TandAb, and sdAb-based	Small-sized structures; single-chain or small dimeric formats; lacking an Fc region	Potential for rapid tissue penetration; no Fc effector function; short *in vivo* half-life	Potentially low molecular stability; risk of aggregation and proteolysis; requires continuous administration or half-life extension strategies	[1,4,14]
Fc-based Symmetric	Fusion	scFv-IgG and sdAb-IgG	Retains IgG bivalency with an additional binding module	Increased avidity; FcRn-mediated half-life extension; tunable effector function	Increased molecular size; possible steric hindrance; greater complexity in expression and purification	[1,4]
	Dual-variable stacking	DVD-Ig	Stacked outer and inner variable domain structure	Enhanced avidity through multivalent binding; improved potential for simultaneous binding	Possible interdomain interference; increased folding complexity; expression optimization required	[4,15,16]
	Tandem Fab	FIT-Ig	IgG-like symmetric bispecific format with Fab modules fused in tandem	High binding stability; enables multivalent binding	Increased molecular size may impose burdens on production and formulation	[4,17,18]
Fc-based Asymmetric	HC heterodimerization	Knob-into-Hole and electrostatic steering IgG	Independent 1 + 1 arm-specific arrangement	Precise dual targeting; readily applicable to affinity asymmetry strategies	Requires optimization of heavy-chain selectivity; minimization of homodimer formation is necessary	[4,19,20,21]
	LC pairing control	Common LC and CrossMab	Structurally minimizes light-chain mispairing	Improved molecular homogeneity; increased functional reproducibility	Increased design complexity; interface optimization required	[4,22,23,24]
Post-assembly	Fab-arm exchange	Controlled Fab-arm exchange	Separates the burden of simultaneous intracellular assembly	High combinatorial accuracy	Process condition control is critical; process consistency during scale-up must be ensured	[25,26]

**Table 2:** Structural platform-dependent valency architectures and their functional consequences.

Structure	Subcategory	Valency	Structural Characteristics	Functional Implications	Design Implications	Reference
Fragment-based	Tandem scFv	Predominantly 1 + 1	Single-chain format; limited potential for additional bivalency	Limited avidity amplification; strong dependence on *K*_D_ and *k*_off_	Increased dependence on intrinsic affinity and binding kinetics; suitability for controlled monovalent engagement	[14,27,28]
	TandAb	2 + 2	Self-dimerization-based multivalent format	Increased rebinding; enhanced avidity	Enhanced retention on target cells when antigen density and molecular geometry permit multivalent engagement	[30]
Fc-based Symmetric	Fusion	Commonly 2 + 2	Intrinsic IgG bivalency with an additional binding module	Selective avidity enhancement; prolonged residence time	Bivalent engagement for both targets and FcRn-based half-life acquisition are possible	[4,6]
	DVD-Ig	2 + 2	Variable domain stacking format	Receptor clustering; enhanced signaling blockade	Suitability for dual receptor blockade strategies	[16,51]
Fc-based Asymmetric	HC heterodimerization	1 + 1	Independent antigen specificity in each arm	Affinity asymmetry; potential CRS modulation	High-affinity tumor-binding arm with low-affinity CD3-binding arm design	[4,52,53]

**Table 3:** Mechanism-dependent epitope geometry and structural design implications in bispecific antibodies.

Mechanistic Context	Representative Structural Formats	Key Geometric Determinants	Functional Consequences	Design Implications	Reference
Immune-cell redirection	Tandem scFv and 1 + 1 or 2 + 1 IgG-like immune cell engagers	Target-epitope proximity to the cell membrane, antigen size, binding-arm orientation, and the resulting intercellular spacing	Determines the organization and stability of the immune synapse and influences target-cell killing	The epitope and molecular format should be selected to support productive immune cell–target cell contact rather than simply to minimize epitope distance	[7,8,55,56,59]
Biparatopic engagement of the same receptor	Biparatopic IgG and DVD-Ig formats	Relative location and orientation of the two epitopes and the receptor arrangement generated after dual engagement	Can induce receptor clustering, internalization, downregulation, or signaling modulation	The epitope pair should be selected according to the receptor organization required for the intended mechanism of action	[49,50,57]
Simultaneous engagement of two different receptors	Asymmetric or multivalent IgG-like bsAbs	Accessibility of both epitopes, inter-receptor distance, binding-arm reach, and orientation of the two binding modules	Can support simultaneous receptor blockade, receptor downmodulation, or coordinated inhibition of multiple signaling pathways	Molecular geometry should permit concurrent target engagement or create the receptor proximity required for the intended functional output	[58,60]

**Table 4:** Mechanism-dependent affinity and kinetic design principles in bispecific antibodies.

Mechanistic Context	Primary Affinity and Kinetic Objective	Relevant Structural and Biological Variables	Functional Consequences	Design Implications and Trade-Offs	Reference
T-cell-engaging bsAbs	Combine sufficient tumor-cell retention with controlled CD3 engagement	Tumor-antigen density, tumor-arm affinity and valency, CD3 affinity and valency, binding-arm arrangement, and epitope geometry	Determines target-dependent cytotoxicity, T-cell activation, cytokine release, and discrimination between tumor and normal cells	Attenuated CD3 affinity can reduce cytokine release while preserving cytotoxicity, but excessive attenuation can reduce activity. Increasing tumor-arm affinity may improve retention but can reduce selectivity for tumor cells over normal cells with lower antigen expression	[8,9,46,48,52,63,64]
FVIIIa-mimetic bsAbs	Support productive and dynamic ternary-complex formation rather than stable cell-surface retention	Arm-specific *K*_D_ and *k*_off_, relative binding stability, target concentration, and spatial compatibility of the bridged factors	One binding arm can contribute more strongly to complex stability, whereas the other can support rapid complex formation and dissociation	The affinity and residence time of each arm should be matched to its role in cofactor-like bridging; maximum affinity toward both targets is not necessarily required	[61,67]
Biparatopic bsAbs targeting the same receptor	Enable simultaneous dual-epitope engagement and the receptor organization required for the intended function	Affinity of each epitope-binding arm, epitope accessibility, valency, receptor density, and epitope geometry	Functional avidity and receptor crosslinking can promote clustering, internalization, downregulation, or signaling modulation	High affinity toward one epitope alone does not ensure the intended receptor response; affinity, valency, and epitope geometry should be optimized together	[49,50,57]
bsAbs targeting two different receptors	Maintain sufficient engagement of both receptors to support coordinated blockade or receptor downmodulation	Affinity of each arm, relative receptor expression, epitope accessibility, arm arrangement, and molecular geometry	Can enable simultaneous signaling inhibition, receptor downmodulation, or co-degradation	The affinity of each arm should be matched to receptor expression and the intended mechanism of action; a universal affinity hierarchy between the two arms cannot be defined	[58,60]

**Table 5:** Format-dependent linker design principles and their functional consequences in bispecific antibodies.

Structural Context	Primary Role of the Linker	Structural Consequence	Functional and Developability Impact	Design Implication	Reference
Tandem scFv and other single-chain scFv formats	Connect VH and VL domains or adjacent binding modules while permitting intramolecular folding and domain movement	Linker length and sequence influence domain mobility, binding-site accessibility, and local steric interference	Can affect antigen binding, aggregation, and susceptibility to proteolysis	Linker sequence and flexibility should be selected according to the connected domains; a single flexible-linker sequence or length cannot be applied universally	[34,35]
Diabody-derived formats, including diabody, scDb, and TandAb	Control intrachain versus interchain variable-domain pairing and the resulting oligomeric assembly	Short VH–VL linkers suppress intrachain pairing and promote interchain association; middle-linker length in scDb influences monomeric or dimeric assembly	Change molecular assembly, valency, stability, and biological activity	Linker length should be matched to the intended pairing mode and oligomeric state	[69,70,71]
Stacked variable-domain formats, including DVD-Ig	Separate outer and inner variable domains and regulate access to the inner binding site	Linker design alters interdomain interference, inner-domain accessibility, and ligand association rate	Can change simultaneous target engagement and the functional contribution of the inner variable domain	Domain order and linker design should be optimized together according to epitope accessibility and the intended binding mechanism	[15,16,41]

**Table 6:** Functional design principles of the Fc region in bispecific antibodies.

Fc-Related Interaction or Structural Element	Major Structural Determinants	Functional Consequence	Design Implications in bsAbs	Reference
FcRn-mediated recycling	Fc amino-acid residues involved in FcRn binding and preservation of the Fc structure	Protects internalized IgG from intracellular degradation and prolongs systemic exposure	FcRn binding can be retained when extended exposure or intermittent dosing is required, even when FcγR- and C1q-mediated activities are attenuated	[72,73,76]
FcγR-mediated cellular effector activity	IgG subclass, Fc amino-acid sequence, and Fc glycosylation, including core fucosylation	Recruits FcγR-expressing immune cells and can promote cellular effector functions such as ADCC	FcγR binding can be retained or enhanced when innate immune-cell recruitment contributes to the intended mechanism, but can be attenuated when the two antigen-binding arms should remain the principal drivers of activity	[72,75,76]
C1q-mediated complement activation	Fc sequence and the spatial organization of antigen-bound Fc regions, including Fc–Fc assembly on the target surface	Promotes C1q recruitment and initiation of the classical complement pathway	C1q interaction can be preserved when complement activation is required, whereas effector-silent Fc variants can be used when complement activity is not part of the intended mechanism	[74,76]
CH3-dependent heavy-chain assembly	Complementary engineering of the CH3–CH3 interface, including steric and electrostatic heterodimerization strategies	Promotes selective pairing of two different heavy chains and reduces undesired homodimer formation	In asymmetric IgG-like bsAbs, CH3-interface design should achieve correct chain pairing while preserving the required FcRn, FcγR, and C1q properties	[19,21,72]

**Table 7:** Representative in silico applications of Rosetta and AlphaFold in bispecific antibody design.

Target Structural Variable	Computational Tool	Analysis/Prediction Elements	Functional Impact	Strategic Meaning	Reference
Heavy chain heterodimerization (CH3–CH3 interface)	Rosetta (multistate design and interface analysis)	Relative interface energy; buried surface area; electrostatic complementarity	Improved chain selectivity; reduced homodimer formation	Assembly stability design for asymmetric Fc-based bsAbs	[43]
Light chain pairing (CH1–CL interface)	Rosetta interface redesign	Interface energy optimization; orthogonal interface design	Reduced incorrect heavy–light chain pairing	Molecular homogeneity improvement in IgG-like bsAbs	[24,101]
Affinity asymmetry (CD3 vs tumor arm)	Rosetta-based CDR/ interface modeling for affinity hypothesis generation	Binding-energy changes from interface alterations	Candidate variants affecting synapse maintenance, activity, and cytokine release risk	Structure–function hypothesis generation and candidate prioritization	[10,52,53,63,64,65]
Epitope geometry (biparatopic, dual receptor)	AlphaFold-Multimer	Domain orientation; epitope distance; steric clash likelihood	Altered simultaneous binding and receptor clustering	Design prioritization for geometry-dependent signaling	[11,12,13,49,57,102,103]
T-cell engager synapse-related arrangement	AlphaFold + structural modeling	Arm orientation; membrane-proximal accessibility	Altered immune cell–tumor cell contact and synapse formation	Early exclusion of functionally unfavorable arrangements	[7,11,12,13,55,56,59,104]
Linker length/rigidity	Structural modeling + energy minimization	Interdomain distance and rotational freedom	Changes in simultaneous binding probability and steric interference	Initial guidance for flexible or semi-rigid linker selection	[10,34,69,70]

## Data Availability

No new data were created or analyzed in this study. Data sharing is not applicable to this article.
